# Supplementary figures and images for: Broad antifungal resistance mediated by RNAi-dependent epimutation in the basal human fungal pathogen Mucor circinelloides
Source: PLoS Genet. 2019 Feb 11;15(2):e1007957. doi: 10.1371/journal.pgen.1007957 (PMC6386414; doi:10.1371/journal.pgen.1007957)

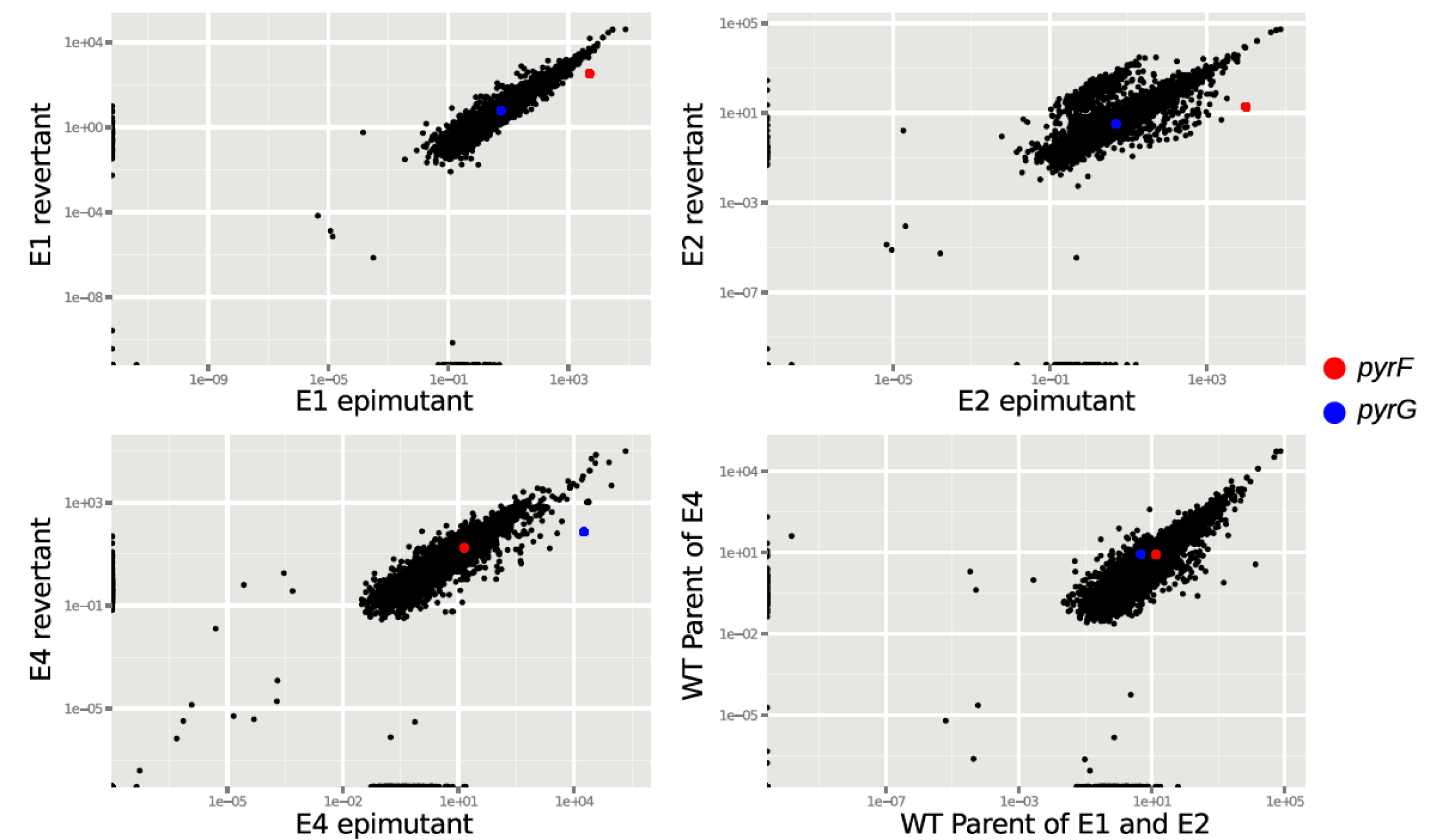

Supplement: S1 Fig — Genome-wide sRNA levels are plotted between two sequenced libraries, with values for one library plotted on the X and the other on the Y. The point representing pyrF is depicted in red and the pyrG is depicted in blue. E1 and E2 are pyrF epimutant strains; E4 is a pyrG epimutant strain. (TIF) [file pgen.1007957.s001.tif]

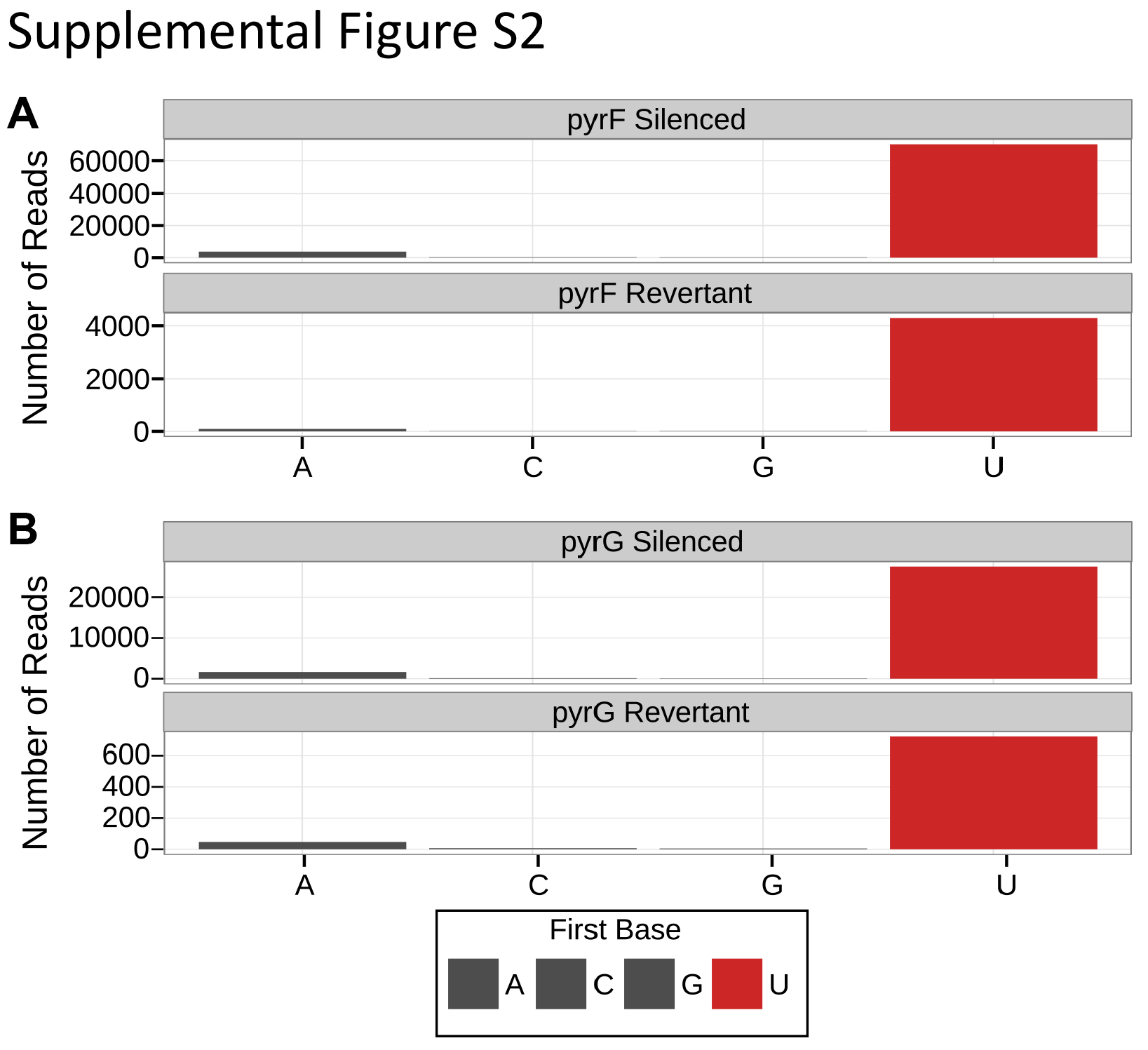

Supplement: S2 Fig — Analysis of the 5’ nucleotide of antisense sRNAs that map to the pyrF and pyrG loci. Data from Fig 3 is replotted here with an expanded y-axis to enable easier comparison of 5’ terminal nucleotides in sRNA from revertant strains. (A) 5’ terminal nucleotides of antisense sRNAs isolated from pyrF epimutant E1 and revertant. (B) 5’ terminal nucleotides of antisense sRNAs isolated from pyrG epimutant E4 and revertant. (TIF) [file pgen.1007957.s002.tif]

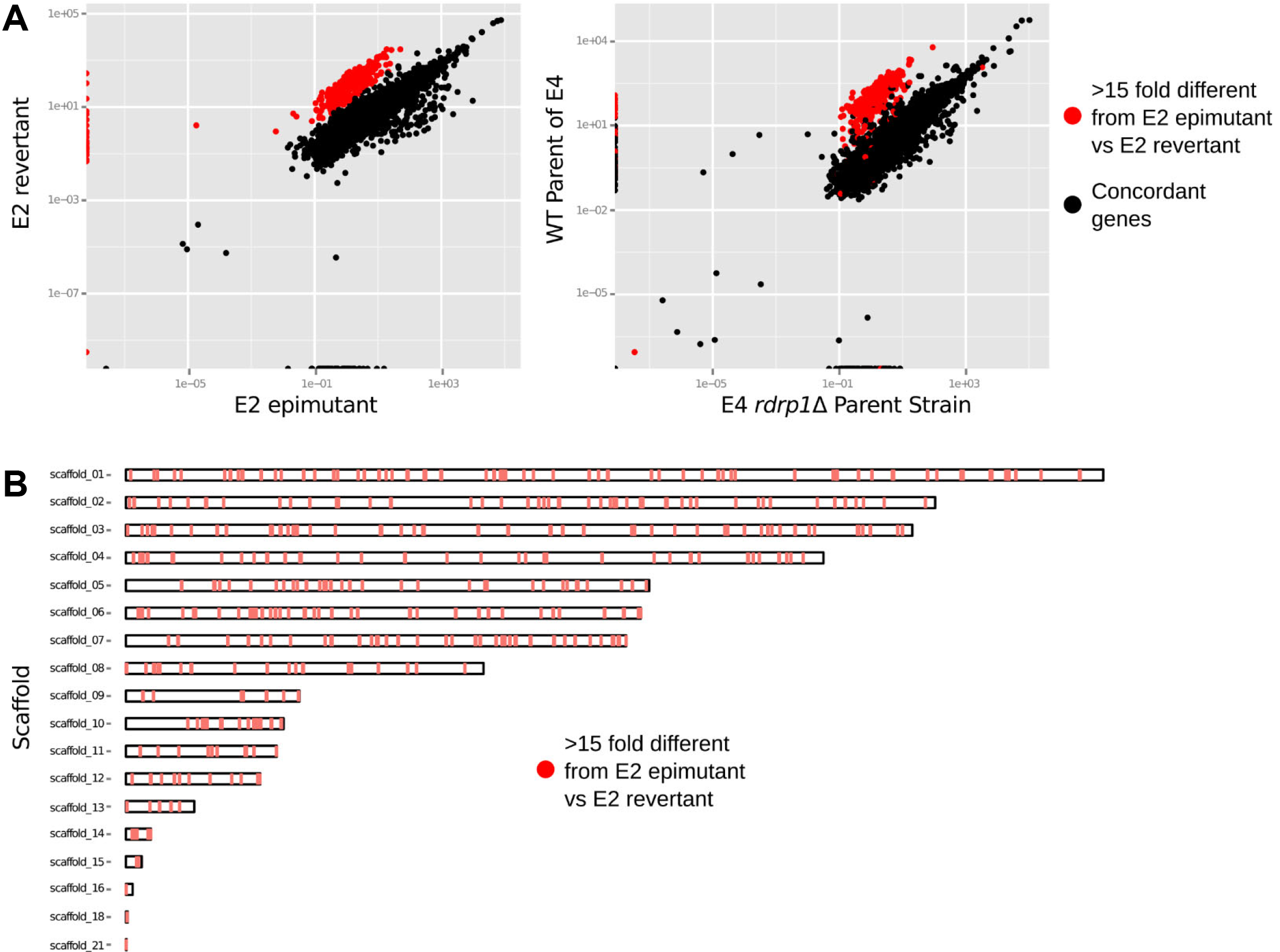

Supplement: S3 Fig — (A) Genome-wide sRNA levels are shown with the gene set that is expressed more than 15-fold higher in the E2 revertant than in the E2 epimutant shaded in red. That same gene set is also shaded in the comparison of the rdrp1Δ parent strain with the WT parent to demonstrate that the same gene set is behaving anomalously in both comparisons. (B) Genes with discordant sRNA expression are shown across the Mucor genome (red bars not to scale relative to scaffold). These genes appear on every scaffold of the genome that is greater than 41 kb in size. (TIF) [file pgen.1007957.s003.tif]

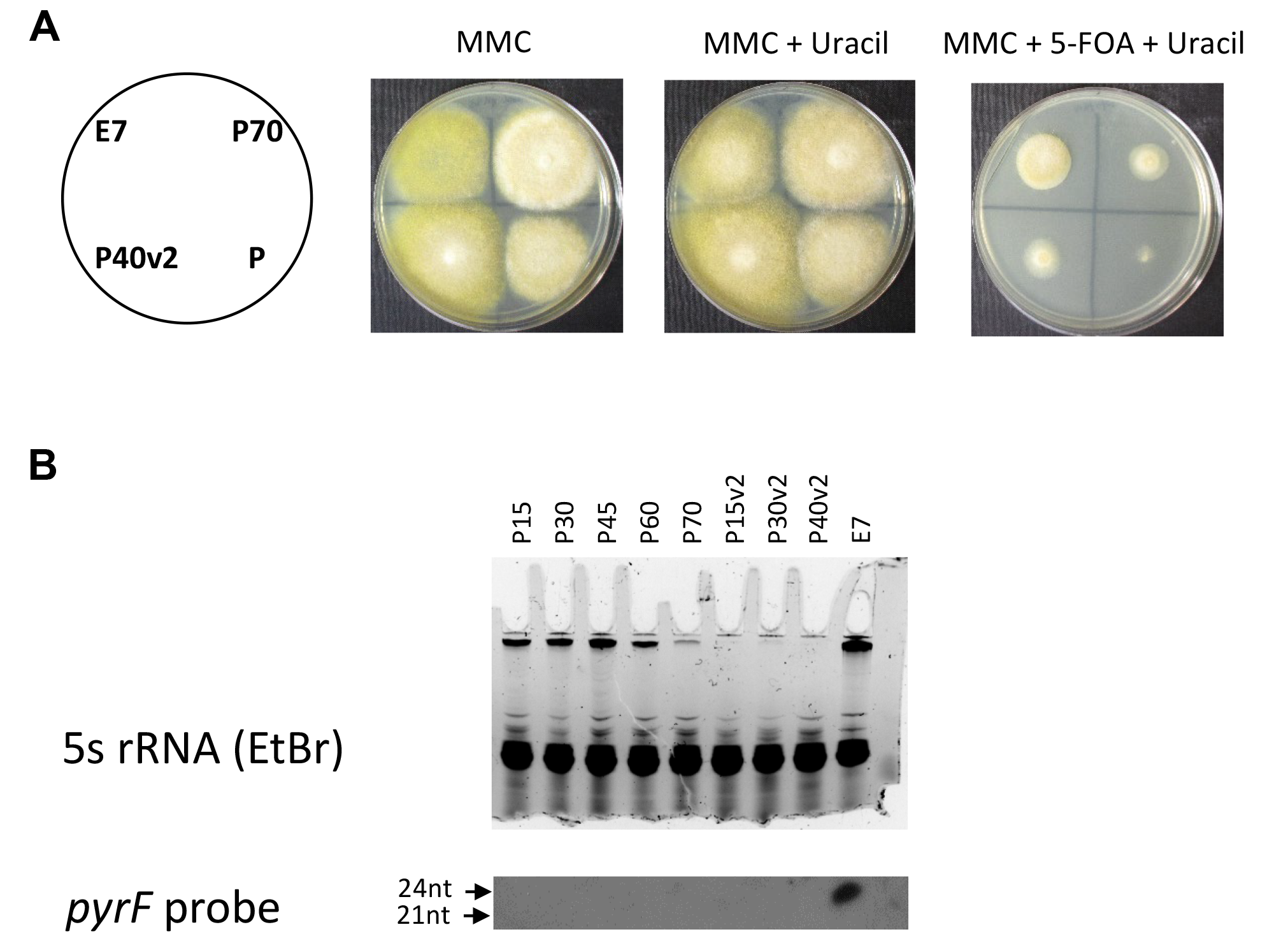

Supplement: S4 Fig — (A) The pyrF epimutant E7 maintains a degree of 5-FOA resistance even after 70 passages without drug selection (P70). An independent repeat of passaging demonstrates continued 5-FOA resistance through 40 passages (P40v2). Strains were grown on MMC media, MMC supplemented with uridine and uracil, and MMC supplemented with 5-FOA, uridine, and uracil. P, rdrp1Δ parental strain (MU419). (B) sRNA hybridization of passaged strains of epimutant E7. Epimutant E7 expresses sRNA against pyrF, but this sRNA is no longer expressed from 15 passages (P15) through 70 passages (P70). Similarly, strains from an independent set of passages demonstrate no sRNA against pyrF at passages 15 (P15v2), 30 (P30v2), or 40 (P40v2). The top portion of the gel was stained with ethidium bromide (EtBr) to visualize the 5S rRNA loading control, after which sRNA was transferred to a membrane for hybridization with an antisense-specific probe against pyrF. (TIF) [file pgen.1007957.s004.tif]
