# Supplementary material for: Broad antifungal resistance mediated by RNAi-dependent epimutation in the basal human fungal pathogen Mucor circinelloides
Source: PLoS Genet. 2019 Feb 11;15(2):e1007957. doi: 10.1371/journal.pgen.1007957 (PMC6386414; doi:10.1371/journal.pgen.1007957)
Supplement: S1 Table — (DOCX) [file pgen.1007957.s005.docx]

**S1 Table. Primers used in this study**

| Name | Sequence | Use |
| --- | --- | --- |
| JOHE41713 | GAGCTGCTGTAAGGCTGGAC | R7B *pyrF* forward |
| JOHE41714 | TGAAATGGGATCCATCAACA | R7B *pyrF* reverse |
| JOHE41962 | AGAGTCACCCATTTTGAGTGC | R7B *pyrF* forward |
| JOHE41963 | TTGCTAGATCCGGGTTTCAC | R7B *pyrF* reverse |
| JOHE42127 | CATTTGGCACCATCATTCAG | Locus-specific amplification of *pyrG* in *rdrp3* mutant strains |
| JOHE42128 | GTTGGGTCGACTGTCGTTTT | Locus-specific amplification of *pyrG* in *rdrp3* mutant strains |
| JOHE43751 | CGTCAGATCGTTCTTGCAGG | Locus-specific amplification of *pyrG* in *rdrp1* mutant strains |
| JOHE43752 | CGGTCTCTCCCCATAACACG | Locus-specific amplification of *pyrG* in *rdrp1* mutant strains |
| JOHE44080 | ATTGGGATGCTGTTGTCCAC | R7B *pyrG* sequencing |
| JOHE42081 | CAAATGACATTAGCCCCTTGA | R7B *pyrG* sequencing |
| JOHE42082 | CGAGGTTGGTCTTCCTCTTG | R7B *pyrG* sequencing |
| JOHE42083 | TGGTCTGGGTTGCCATAGAT | R7B *pyrG* sequencing |
| JOHE41665 | TTGAGTGTGCGGAGATCTTG | 1006PhL *pyrF* forward |
| JOHE41666 | TGACCTCACGTGGTTGATCT | 1006PhL *pyrF* reverse |
| JOHE41667 | CGCTGGATCTGGGTGATATT | 1006PhL *pyrG* forward |
| JOHE41668 | CCTGCCTTAACTCCCATCAA | 1006PhL *pyrG* reverse |
| JOHE41669 | CGAGGACTTTGACCGTGATT | 1006PhL *pyrG* sequencing |
| JOHE38278 | GAGGAATGAGACCGGGGTAACCAC | 24mer for size standard on sRNA blots |
| JOHE42163 | AGATCCACGATCACGAGATGA | 21mer for size standard on sRNA blots |
| JOHE42212 | TAATACGACTCACTATAGGGTTGAGTGTGCGGAGATCTTG | T7 promoter and 1006PhL *pyrF* forward. For sRNA antisense probe synthesis combined with JOHE41666 |
| JOHE42354 | TAATACGACTCACTATAGGGCGCTGGATCTGGGTGATATT | T7 promoter and 1006PhL *pyrG* forward. For sRNA antisense probe synthesis combined with JOHE41668 |
| JOHE42440 | TAATACGACTCACTATAGGGAGAGTCACCCATTTTGAGTGC | T7 promoter and *pyrF* forward primer, R7B. For sRNA antisense probe synthesis combined with JOHE41963. |
| JOHE42441 | TAATACGACTCACTATAGGGCGAATGGAAAGTGAGTGGGT | T7 promoter and *pyrG* forward primer, R7B. For sRNA antisense probe synthesis combined with JOHE20867. |
| JOHE20867 | GTACACTGGCCATGCTATCG | *pyrG* reverse primer, R7B |
| JOHE37682 | TAATACGACTCACTATAGGGAGCTACGGCCATACAATGTTG | T7 promoter and 5s rRNA, for amplification of the 5S rRNA probe |
| JOHE37683 | TAATACGACTCACTATAGGGGAACTACAGCAACCAGTATTCCCA | T7 promoter and 5s rRNA, for amplification of the 5S rRNA probe |
| JOHE42636 | ACCGCAAGGAAAAGAAGGAT | R7B *pyrF* qRT-PCR |
| JOHE42637 | CAAGGACACCAGCAAGTTGA | R7B *pyrF* qRT-PCR |
| JOHE44065 | TTGATGGAGCGTAAGCAATC | R7B *pyrG* qRT-PCR |
| JOHE44066 | AGCAACCAAATCGTGGTCA | R7B *pyrG* qRT-PCR |
| JOHE24077 | AAGCCCAATCCAAGAGAGGT | R7B actin qRT-PCR |
| JOHE24078 | GCCTCAGTCAAGAGGACAGG | R7B actin qRT-PCR |
