# Supplementary material for: Broad antifungal resistance mediated by RNAi-dependent epimutation in the basal human fungal pathogen Mucor circinelloides
Source: PLoS Genet. 2019 Feb 11;15(2):e1007957. doi: 10.1371/journal.pgen.1007957 (PMC6386414; doi:10.1371/journal.pgen.1007957)
Supplement: S2 Table — (DOCX) [file pgen.1007957.s006.docx]

**S2 Table. Strains generated in this study**

| **Strain** | **#** | **Epimutant** | **Genetic background** | **Mutation/cause of resistance** |
| --- | --- | --- | --- | --- |
| ***rdrp3*∆** | **1** |  | MU500 | Unknown; no mutations or *pyrF/pyrG* sRNA found |
|  | **2** |  | MU500 | Unknown; no mutations or *pyrF/pyrG* sRNA found |
|  | **3** |  | MU500 | Unknown; no mutations or *pyrF/pyrG* sRNA found |
|  | **4** |  | MU500 | Unknown; no mutations or *pyrF/pyrG* sRNA found |
|  | **5** |  | MU500 | Unknown; no mutations or *pyrF/pyrG* sRNA found |
|  | **6** |  | MU500 | Unknown; no mutations or *pyrF/pyrG* sRNA found |
|  | **7** |  | MU500 | Unknown; no mutations or *pyrF/pyrG* sRNA found |
|  | **8** |  | MU500 | Unknown; no mutations or *pyrF/pyrG* sRNA found |
|  | **9** |  | MU440 | Unknown; no mutations or *pyrF/pyrG* sRNA found |
|  | **10** |  | MU440 | Unknown; no mutations or *pyrF/pyrG* sRNA found |
|  | **11** |  | MU440 | Unknown; no mutations or *pyrF/pyrG* sRNA found |
|  | **12** | **E1** | MU439 | ***pyrF* epimutant** |
|  | **13** |  | MU439 | Unknown; no mutations or *pyrF/pyrG* sRNA found |
|  | **17** | **E2** | MU439 | ***pyrF* epimutant** |
| ***rdrp1*∆** | **1** | **E6** | MU419 | ***pyrF* epimutant** |
|  | **2** |  | MU419 | *pyrG* G413A; exon splice site |
|  | **3** |  | MU419 | *pyrG* G413A; exon splice site |
|  | **4** |  | MU419 | *pyrG* G413A; exon splice site |
|  | **5** |  | MU419 | *pyrG* G413A; exon splice site |
|  | **6** |  | MU419 | *pyrG* G413A; exon splice site |
|  | **7** |  | MU419 | *pyrG* G413A; exon splice site |
|  | **8** |  | MU419 | *pyrG* G413A; exon splice site |
|  | **9** | **E7** | MU419 | ***pyrF* epimutant** |
|  | **10** |  | MU419 | *pyrG* G413A; exon splice site |
|  | **11** |  | MU419 | *pyrG* G413A; exon splice site |
|  | **12** | **E3** | MU419 | ***pyrF* epimutant** |
|  | **13** |  | MU419 | Unknown; no mutations or sRNA found |
|  | **14** | **E4** | MU419 | ***pyrG* epimutant** |
|  | **15** |  | MU419 | Unknown; no mutations or sRNA found |
|  | **16** |  | MU419 | *pyrG* G413A; exon splice site |
|  | **17** |  | MU419 | *pyrG* G413A; exon splice site |
|  | **18** |  | MU419 | *pyrG* G413A; exon splice site |
|  | **19** |  | MU419 | *pyrG* G413A; exon splice site |
|  | **20** |  | MU419 | *pyrG* G413A; exon splice site |
|  | **21** |  | MU419 | *pyrG* G413A; exon splice site |
|  | **22** |  | MU419 | *pyrG* G413A; exon splice site |
|  | **23** | **E5** | MU419 | ***pyrF* epimutant** |
|  | **24** |  | MU419 | *pyrG* G413A; exon splice site |
|  | **25** |  | MU419 | *pyrG* G413A; exon splice site |
|  | **26** |  | MU419 | *pyrG* G413A; exon splice site |
|  | **27** |  | MU419 | *pyrG* G413A; exon splice site |
| ***r3b2*∆** | **1** |  | MU412 | *pyrG* G413A; exon splice site |
|  | **2** |  | MU412 | *pyrG* G413A; exon splice site |
|  | **3** |  | MU412 | *pyrG* G413A; exon splice site |
|  | **4** |  | MU412 | Unknown; no mutations or sRNA found |
|  | **5** |  | MU412 | *pyrG* G413A; exon splice site |
|  | **6** |  | MU412 | *pyrG* G413A; exon splice site |
|  | **7** |  | MU412 | *pyrG* G413A; exon splice site |
|  | **8** |  | MU412 | *pyrG* G413A; exon splice site |
|  | **9** |  | MU412 | *pyrG* G413A; exon splice site |
|  | **10** |  | MU412 | *pyrG* G413A; exon splice site |
|  | **11** |  | MU412 | *pyrG* G413A; exon splice site |
|  | **12** |  | MU412 | *pyrG* G413A; exon splice site |
|  | **13** |  | MU412 | *pyrG* G413A; exon splice site |
|  | **14** |  | MU412 | Unknown; no mutations or sRNA found |
| **R7B** | **1** |  | R7B | Unknown; no mutations or *pyrF/pyrG* sRNA found |
|  | **2** |  | R7B | Unknown; no mutations or *pyrF/pyrG* sRNA found |
| **1006PhL** | **1** |  | 1006PhL | Unknown; no mutations or *pyrF/pyrG* sRNA found |
|  | **2** |  | 1006PhL | Unknown; no mutations or *pyrF/pyrG* sRNA found |
|  | **3** |  | 1006PhL | Unknown; no mutations or *pyrF/pyrG* sRNA found |
|  | **4** |  | 1006PhL | Unknown; no mutations or *pyrF/pyrG* sRNA found |
|  | **5** |  | 1006PhL | Unknown; no mutations or *pyrF/pyrG* sRNA found |
|  | **6** |  | 1006PhL | Unknown; no mutations or *pyrF/pyrG* sRNA found |
|  | **7** |  | 1006PhL | Unknown; no mutations or *pyrF/pyrG* sRNA found |
|  | **8** |  | 1006PhL | Unknown; no mutations or *pyrF/pyrG* sRNA found |
|  | **9** |  | 1006PhL | Unknown; no mutations or *pyrF/pyrG* sRNA found |
|  | **10** |  | 1006PhL | Unknown; no mutations or *pyrF/pyrG* sRNA found |
|  | **11** |  | 1006PhL | *pyrF* C535T (nonsense mutation) * |
|  | **12** |  | 1006PhL | *pyrF* C535T (nonsense mutation) * |
|  | **13** |  | 1006PhL | *pyrF* C535T (nonsense mutation) * |
|  | **14** |  | 1006PhL | *pyrF* C535T (nonsense mutation) * |
|  | **15** |  | 1006PhL | *pyrF* C535T (nonsense mutation) * |
|  | **16** |  | 1006PhL | *pyrF* C535T (nonsense mutation) * |
|  | **17** |  | 1006PhL | *pyrF* C535T (nonsense mutation) * |
|  | **18** |  | 1006PhL | Unknown; no mutations or *pyrF/pyrG* sRNA found |
|  | **19** |  | 1006PhL | Unknown; no mutations or *pyrF/pyrG* sRNA found |
|  | **20** |  | 1006PhL | Unknown; no mutations or *pyrF/pyrG* sRNA found |
|  | **21** |  | 1006PhL | Unknown; no mutations or *pyrF/pyrG* sRNA found |
|  | **22** |  | 1006PhL | Unknown; no mutations or *pyrF/pyrG* sRNA found |
|  | **23** |  | 1006PhL | *pyrF* C535T (nonsense mutation) * |
|  | **24** |  | 1006PhL | *pyrF* C535T (nonsense mutation) * |
|  | **25** |  | 1006PhL | *pyrF* C535T (nonsense mutation) * |
|  | **26** |  | 1006PhL | *pyrF* C535T (nonsense mutation) * |
|  | **27** |  | 1006PhL | Unknown; no mutations or *pyrF/pyrG* sRNA found |
|  | **28** |  | 1006PhL | Unknown; no mutations or *pyrF/pyrG* sRNA found |
|  | **29** |  | 1006PhL | Unknown; no mutations or *pyrF/pyrG* sRNA found |
|  | **30** |  | 1006PhL | Unknown; no mutations or *pyrF/pyrG* sRNA found |
|  | **31** |  | 1006PhL | Unknown; no mutations or *pyrF/pyrG* sRNA found |
|  | **32** |  | 1006PhL | Unknown; no mutations or *pyrF/pyrG* sRNA found |
|  | **33** |  | 1006PhL | Unknown; no mutations or *pyrF/pyrG* sRNA found |
|  | **34** |  | 1006PhL | Unknown; no mutations or *pyrF/pyrG* sRNA found |
|  | **35** |  | 1006PhL | Unknown; no mutations or *pyrF/pyrG* sRNA found |
|  | **36** |  | 1006PhL | Unknown; no mutations or *pyrF/pyrG* sRNA found |
|  | **37** |  | 1006PhL | Unknown; no mutations or *pyrF/pyrG* sRNA found |
|  | **38** |  | 1006PhL | Unknown; no mutations or *pyrF/pyrG* sRNA found |
|  | **39** |  | 1006PhL | Unknown; no mutations or *pyrF/pyrG* sRNA found |
|  | **40** |  | 1006PhL | Unknown; no mutations or *pyrF/pyrG* sRNA found |
|  | **41** |  | 1006PhL | Unknown; no mutations or *pyrF/pyrG* sRNA found |
|  | **42** |  | 1006PhL | Unknown; no mutations or *pyrF/pyrG* sRNA found |
|  | **43** |  | 1006PhL | Unknown; no mutations or *pyrF/pyrG* sRNA found |
|  | **44** |  | 1006PhL | *pyrF* C535T (nonsense mutation) * |
|  | **45** |  | 1006PhL | *pyrF* C535T (nonsense mutation) * |
|  | **46** |  | 1006PhL | *pyrF* C535T (nonsense mutation) * |
|  | **47** |  | 1006PhL | *pyrF* C535T (nonsense mutation) * |
|  | **48** |  | 1006PhL | *pyrF* C535T (nonsense mutation) * |
|  | **49** |  | 1006PhL | *pyrF* C535T (nonsense mutation) * |
|  | **50** |  | 1006PhL | *pyrF* C535T (nonsense mutation) * |
|  | **51** |  | 1006PhL | *pyrF* C535T (nonsense mutation) * |
|  | **52** |  | 1006PhL | *pyrF* C535T (nonsense mutation) * |
|  | **53** |  | 1006PhL | *pyrF* T288 1bp deletion |
|  | **54** |  | 1006PhL | *pyrF* C535T (nonsense mutation) * |
|  | **55** |  | 1006PhL | Unknown; no mutations or *pyrF/pyrG* sRNA found |
|  | **56** |  | 1006PhL | *pyrF* C535T (nonsense mutation) * |
|  | **57** |  | 1006PhL | *pyrF* C535T (nonsense mutation) * |
|  | **58** |  | 1006PhL | *pyrF* C535T (nonsense mutation) * |
|  | **59** |  | 1006PhL | *pyrF* C535T (nonsense mutation) * |
|  | **60** |  | 1006PhL | *pyrF* C535T (nonsense mutation) * |
|  | **61** |  | 1006PhL | *pyrF* C535T (nonsense mutation) * |
|  |  |  |  |  |

* The prevalence of this single mutation in the 1006PhL background suggests these could potentially be sibling strains derived from a mutation present in the original population.
